# Elevated Circulating Sclerostin Concentrations in Individuals With High Bone Mass, With and Without *LRP5* Mutations

**DOI:** 10.1210/jc.2013-3958

**Published:** 2014-02-25

**Authors:** Celia L. Gregson, Kenneth E. S. Poole, Eugene V. McCloskey, Emma L. Duncan, Jörn Rittweger, William D. Fraser, George Davey Smith, Jonathan H. Tobias

**Affiliations:** Musculoskeletal Research Unit (C.L.G., J.H.T.), School of Clinical Sciences, University of Bristol, Bristol BS10 5NB, United Kingdom; Medical Research Council (MRC) Lifecourse Epidemiology Unit (C.L.G.), University of Southampton, Southampton, SO16 6YD United Kingdom; Department of Medicine (K.E.S.P.), University of Cambridge, Cambridge, CB2 0SP United Kingdom; Metabolic Bone Centre (E.V.M.), Sheffield University, Sheffield, S3 7HF United Kingdom; Human Genetics Group (E.L.D.), University of Queensland Diamantina Institute, Brisbane, Australia; Department of Endocrinology (E.L.D), Royal Brisbane and Women's Hospital, Brisbane, Australia; Institute of Aerospace Medicine (J.R.), German Aerospace Center (Deutschen Zentrums fuür Luft- und Raumfahrt), Cologne, Germany; Institute for Biomedical Research into Human Movement and Health Research Institute (J.R.), Manchester Metropolitan University, Manchester, M1 5GD United Kingdom; Department of Medicine (W.D.F.), Norwich Medical School, University of East Anglia, Norwich, NR4 7TJ United Kingdom; and MRC Integrative Epidemiology Unit (G.D.S.), School of Social and Community Based Medicine, University of Bristol, Bristol, BS8 2BN United Kingdom

## Abstract

**Context:**

The role and importance of circulating sclerostin is poorly understood. High bone mass (HBM) caused by activating *LRP5* mutations has been reported to be associated with increased plasma sclerostin concentrations; whether the same applies to HBM due to other causes is unknown.

**Objective:**

Our objective was to determine circulating sclerostin concentrations in HBM.

**Design and Participants:**

In this case-control study, 406 HBM index cases were identified by screening dual-energy x-ray absorptiometry (DXA) databases from 4 United Kingdom centers (n = 219 088), excluding significant osteoarthritis/artifact. Controls comprised unaffected relatives and spouses.

**Main measures:**

Plasma sclerostin; lumbar spine L1, total hip, and total body DXA; and radial and tibial peripheral quantitative computed tomography (subgroup only) were evaluated.

**Results:**

Sclerostin concentrations were significantly higher in both *LRP5* HBM and non-*LRP5* HBM cases compared with controls: mean (SD) 130.1 (61.7) and 88.0 (39.3) vs 66.4 (32.3) pmol/L (both *P* < .001, which persisted after adjustment for a priori confounders). In combined adjusted analyses of cases and controls, sclerostin concentrations were positively related to all bone parameters found to be increased in HBM cases (ie, L1, total hip, and total body DXA bone mineral density and radial/tibial cortical area, cortical bone mineral density, and trabecular density). Although these relationships were broadly equivalent in HBM cases and controls, there was some evidence that associations between sclerostin and trabecular phenotypes were stronger in HBM cases, particularly for radial trabecular density (interaction *P* < .01).

**Conclusions:**

Circulating plasma sclerostin concentrations are increased in both *LRP5* and non-*LRP5* HBM compared with controls. In addition to the general positive relationship between sclerostin and DXA/peripheral quantitative computed tomography parameters, genetic factors predisposing to HBM may contribute to increased sclerostin levels.

Sclerostin is an endogenous, osteocyte-derived, soluble inhibitor of canonical Wnt signaling and a potent inhibitor of osteoblastic bone formation. Despite associations with a range of factors, its role and importance is poorly understood. In contrast, protein function and expression analyses have advanced understanding of sclerostin's paracrine effects. However, although circulating sclerostin correlates with bone marrow plasma sclerostin, the extent to which plasma sclerostin leakage reflects underlying bone biology is unclear (1). Hence, studying plasma sclerostin in a wide range of bone disorders is desirable. Sclerostin concentrations are known to increase with age, immobility, weight loss, menopause, type 2 diabetes mellitus, and denosumab treatment and are greater in men than women (2–11). Estrogen replacement, PTH therapy, and physical activity decrease sclerostin in postmenopausal women (1, 3, 12), whereas bisphosphonates have variable effects (7, 13, 14). Oral glucocorticoids decrease sclerostin concentrations acutely, potentially through osteocyte apoptosis (15).

Sclerostin deficiency, occurring in sclerosteosis (OMIM 269500) and Van Buchem's disease (OMIM 239100), leads to widespread increased bone mineral density (BMD) and a characteristic skeletal dysplasia including fracture resistance (16–18). Heterozygous carriers have high bone mass (HBM) and fracture resistance but an otherwise normal phenotype (19), hence current efforts to develop sclerostin antibodies as a novel anabolic osteoporosis treatment. In rodents, in response to mechanical loading (mechanotransduction), osteocytic sclerostin secretion is reduced, alleviating inhibition of osteoblast activity, increasing bone formation and BMD (20, 21). In humans, raised sclerostin in response to immobility points toward a similar effect (8, 9, 11). However, the total amount of bone may determine plasma sclerostin concentrations because, in the general population, sclerostin is positively related to total-body (TB) BMD, particularly in older individuals, and inversely related to bone turnover in men and pre- and postmenopausal women (3, 4, 22). Recently, sclerostin has been positively associated with several microarchitectural parameters including trabecular density, assessed by high-resolution peripheral quantitative computed tomography (pQCT) (23), and cortical volumetric BMD and area, by pQCT (24).

Elevated sclerostin concentrations have also been reported in an HBM family with a *T253I* mutation in the Wnt pathway regulator low-density lipoprotein receptor-related protein 5 (*LRP5*) (25). Although this may reflect an effect of the mutation on sclerostin metabolism, associations between bone mass, microarchitecture, and sclerostin may equally be responsible. Consistent with the latter suggestion, pQCT analysis of a phenotypically similar HBM population revealed differences in a number of microarchitectural parameters, previously related to sclerostin in the general population, including increased trabecular density and cortical volumetric BMD and area (23, 24, 26). These potential relationships may be complicated further as sclerostin has also been positively associated with fat mass (FM) (27), as has HBM (28); hence, sclerostin-bone relationships may be confounded by adiposity.

We planned to improve understanding of the relationships between bone and circulating sclerostin by examining the rare and extreme HBM phenotype. We aimed to determine whether 1) sclerostin concentrations are elevated in HBM, 2) if any observed differences are explained by *LRP5* HBM mutations, and 3) any differences reflect an altered relationship between sclerostin and bone parameters in HBM individuals, taking into account established confounding factors.

## Subjects and Methods

### Participant recruitment

The HBM study is a United Kingdom-based multicenter observational study of adults with unexplained HBM. At 4 of our largest study centers, 406 HBM index cases were identified by screening National Health Service (NHS) dual-energy x-ray absorptiometry (DXA) databases (n = 219 088), excluding scans with significant osteoarthritis and/or other causes of raised BMD (eg, surgical metalwork, Paget's disease, and metastases). Full details of DXA database screening and participant recruitment have previously been reported (29). In brief, HBM was defined as 1) both L1 Z-score ≥+3.2 and total hip Z-score ≥+1.2 or 2) both total hip Z-score ≥+3.2 and L1 Z-score ≥+1.2. The L1 lumbar vertebra was used because, in contrast to lower lumbar levels, it was not associated with the presence of lumbar spine osteoarthritis assessed on DXA images (29). Index cases passed on study invitations to their first-degree relatives and spouses/partners. Relatives/spouses with HBM in turn passed on invitations to their first-degree relatives/spouses. HBM was defined among spouses as per index cases and among first-degree relatives as summed L1 Z-score plus total hip Z-score of ≥+3.2, reflecting an established family history of HBM. Family-based controls comprised unaffected relatives and spouses. All participants were clinically assessed by one doctor using a standardized structured history and examination questionnaire, after which TB DXA scans and (nonfasted) phlebotomy were performed. None had a history of parathyroid disease.

Subsequently, current and lifetime physical activity (PA) was measured by a short (10-minute) postal questionnaire (prepaid reply envelope, sent up to 3 times) that included 1) the short last 7 days self-administered international PA questionnaire (IPAQ 2002, http://www.ipaq.ki.se/ipaq.htm (30, 31) and (ii) a historical PA questionnaire (32–34). 86.5% completed PA questionnaires: those who did not respond had similar anthropometric characteristics to those who did (data not shown).

Recruitment ran from September 2008 until April 2010. Written informed consent was collected for all in line with the Declaration of Helsinki (35). Participants were excluded if <18 years of age, pregnant, or unable to provide written informed consent for any reason. This study was approved by the Bath Multicenter Research Ethics Committee and at each NHS Local Research Ethics Committee.

### Sclerostin and bone turnover markers

Two nonfasted EDTA samples were collected and plasma separated and frozen within 4 hours to −80°C. Sclerostin concentrations were measured using ELISA (BI-20442; Biomedica) (detection limit 3.6 pmol/L) (standard range 0–80 pmol/L). Bone formation (procollagen type 1 amino-terminal propeptide [PINP], and total osteocalcin) and resorption (β-C-telopeptides of type I collagen [CTX]) markers were also measured. All had inter- and intra-assay coefficients of variation <6.0% across the assay working ranges. Electrochemiluminescence immunoassays (Roche Diagnostics) were used to measure plasma concentrations of PINP, osteocalcin, and CTX (detection limits 4.0, 0.6, and 0.01 μg/L, respectively).

### DXA measurements

DXA scans were performed using either GE Lunar Prodigy DXA (software version 13.2; GE Healthcare) in Birmingham, Cambridge, and Hull or Hologic Discovery/W DXA (Apex software version 3.0; Hologic Inc) in Sheffield. All scans were acquired and analyzed according to each manufacturer's standard scanning and positioning protocols. TB BMD and FM were measured, together with L1 and total-hip BMD. Known differences in calibration exist between Hologic and GE for all scan types (36, 40). For lumbar spine and hip scans, systematic bias was limited by converting all measures to standardized BMD (38, 39). For TB, systematic differences were limited using cross-calibration equations for all bone and soft tissue regions of interest (40). Full details have previously been reported, including quality control checks and grading of TB scans for metallic artifacts (28). Because only 330 (59.5%) of the original multicenter study population (29) had TB DXA scans performed, the principal characteristics of individuals who received a TB DXA scan were compared with those who did not. No differences were observed in weight, height, sex, age, or ethnicity (data not shown).

### pQCT measurements

At our largest study center, with the necessary equipment, pQCT scanning was performed at the distal and midshaft of the tibia (4% and 66% from distal endplate) and radius (4% and 60%) in the nondominant lower and upper limbs, respectively, using a Stratec XCT2000L (Stratec Medizintechnik) with voxel size 0.5 mm, CT speed 30 mm/s, and XCT software version 5.50d; details have been previously described in full (26). The initial frontal scout view determined a distal endplate reference line. Cortical bone was defined using a threshold ≥650 mg/cm^3^ (optimal for bone geometry) (41). Trabecular bone was identified by elimination of cortical bone, and therefore, trabecular density was defined as <650 mg/cm^3^. Cortical parameters were measured: cortical BMD, total bone area (BA) (ie, total bone cross-section, reflecting periosteal expansion), cortical BA (reflecting combined periosteal and endosteal expansion). Strength strain index (SSI) was calculated according to Stratec's manual: SSI = SM × (cortical BMD [mg/cm^3^]/1200 mg/cm^3^), where 1200 mg/cm^3^ represents normal bone physiological density and SM (section modulus) = CSMI/periosteal radius, where CSMI (cross-sectional moment of inertia [cm^4^]) = [Pi] (periosteal radius^4^ − endosteal radius^4^)/4) (42).

### Statistical methods

Descriptive statistics are presented as mean (95% confidence interval [CI]) for continuous and count (percentages) for categorical data. Analyses comparing 2 continuous variables are presented as β-coefficients and 95% CIs for standardized outcomes. Linear regression was used to analyze continuous variables, using random-effects models to allow for the lack of statistical independence due to within-family clustering of environmental factors and shared genotypes. Age, gender, historical/current PA, height, TB FM, menopausal status, and estrogen replacement therapy in women (an established regulator of sclerostin) (12), were considered a priori confounders of associations between HBM status and sclerostin, DXA, and bone turnover parameters.

Further potential confounders included history of malignancy, diabetes mellitus, glucocorticoid (current/previous/never use), antiresorptive medication use (7, 13–15). Bone density and microarchitecture analyses were stratified to assess interactions by HBM case/control status. Data were managed using Microsoft Access (data entry checks; error rate <0.12%) and analyzed using Stata release 12 statistical software (StataCorp).

## Results

### Participant characteristics

In total, 202 HBM cases (151 index cases, 49 affected relatives, and 2 affected spouses) and 123 family controls (87 unaffected relatives and 36 unaffected spouses) were assessed. HBM cases (age range 26–90 years) were older than family controls (19–88 years) and more commonly female, postmenopausal, and had used estrogen replacement (Table 1). Only 4 HBM cases were not of white European origin.

**Table 1.** Clinical Characteristics of HBM Cases and Family Controls^a^

	HBM Cases (n = 202)	Controls (n = 123)	*P* Value^b^
Mean (SD)			
Age, y	61.4 (13.6)	55.2 (16.3)	<.001
Height, cm	166.6 (9.2)	171.6 (10.6)	<.001
Weight, kg	85.3 (17.4)	84.0 (17.4)	.784
BMI, kg/m^2^	30.7 (5.8)	28.4 (5.0)	.001
TB LM, kg	46.8 (10.2)	51.5 (11.3)	<.001
TB FM, kg	35.7 (12.5)	30.0 (11.3)	<.001
n (%)			
Female	153 (76.5)	55 (44.7)	<.001
Postmenopausal	127 (83.0)	29 (52.7)	<.001
Estrogen replacement use (ever)	77 (53.1)	9 (18.4)	<.001
Previous fracture (ever)	75 (37.5)	61 (49.6)	.033
Diabetes mellitus	20 (10.0)	10 (8.1)	.574
Current/previous glucocorticoid use	49 (24.5)	19 (15.5)	.053
Malignancy (ever)	31 (15.5)	7 (5.7)	.008
Current PA (IPAQ) (n = 290)			
Low	28 (15.4)	14 (13.0)	
Moderate	71 (39.0)	41 (38.0)	.791
High	83 (45.6)	53 (49.1)	
Historical PA score (n = 288)			
Very low (0–4)	21 (11.7)	13 (12.0)	
Low (5–7)	34 (18.9)	27 (25.0)	
Moderate (8–10)	37 (20.6)	26 (24.1)	.369
High (11–14)	45 (25.0)	17 (15.7)	
Very high (15–24)	43 (23.9)	25 (23.2)	

Abbreviations: BMI, body mass index; IPAQ, International Physical Activity Questionnaire; LM, lean mass.

a No individuals had hypercalcemia.

b Unadjusted *P* value from regression model accounting for within-family clustering. Only 9 HBM cases and 2 controls had ever used antiresorptive medication.

### Plasma sclerostin concentrations

As expected, sclerostin concentrations were strongly associated with age in both HBM cases (unadjusted standardized β per year increase in age 0.03 [0.02, 0.04], *P* < .001) and controls (0.02 [0.01, 0.03], *P* < .001) to a similar degree (interaction *P* = .48). Sclerostin concentrations (mean [SD]) were higher in males than females in both HBM cases (112.5 [46.8] vs 80.9 [33.7] pmol/L, *P* < .001) and controls (72.8 [37.2] vs 58.6 [23.1] pmol/L, *P* = .042), without evidence of interaction. Sclerostin concentrations were independent of bone turnover markers (overall and in men, women, HBM cases, and controls) and TB FM (data not shown).

Unadjusted sclerostin concentrations were significantly higher among HBM cases compared with controls (Table 2). These differences were maintained after adjustment for a priori confounders, ie, age, gender, historical/current PA, height, TB FM, and in women years since menopause and estrogen replacement therapy. Additional adjustment for diabetes mellitus, malignancy, and glucocorticoid and antiresorptive use did not influence these findings (Supplemental Table 1, published on The Endocrine Society's Journals Online website at http://jcem.endojournals.org).

**Table 2.** DXA and pQCT Measurements in HBM Cases Compared With Family Controls^a^

	HBM Mean (SD)	Control Mean (SD)	Unadjusted Mean Difference (95%CI)	Unadjusted *P* Value	Adjusted Mean Difference^b^ (95% CI)	Adjusted *P* Value^b^
Sclerostin,^c^ pmol/L	89.6 (40.7)^d^	66.4 (32.3)	21.9 (13.6, 30.1)	<.001	23.5 (14.5, 32.4)	<.001
DXA (n = 323)						
L1 sBMD, g/cm^2^	1.40 (0.16)	1.08 (0.16)	0.32 (0.29, 0.36)	<.001	0.35 (0.32, 0.39)	<.001
Total Hip sBMD, g/cm^2^	1.25 (0.18)	0.99 (0.14)	0.25 (0.21, 0.28)	<.001	0.29 (0.25, 0.32)	<.001
TB BMD,^e^ g/cm^2^	1.34 (0.13)	1.22 (0.12)	0.11 (0.09, 0.14)	<.001	0.16 (0.13, 0.18)	<.001
Tibia pQCT (n = 156)						
Total BA, mm^2^	633.5 (98.4)	653.3 (111.0)	−20.3 (−53.4, 12.8)	.229	21.5 (−7.33, 50.3)	.144
cBMD, mg/cm^3^	1127.7 (33.2)	1111.4 (51.9)	16.2 (2.85, 29.6)	.017	18.5 (3.44, 33.6)	.016
Cortical BA, mm^2^	337.6 (55.3)	325.2 (67.6)	12.4 (−6.95, 31.7)	.209	33.4 (20.3, 46.4)	<.001
SSI, mm^3^	1651.0 (363.1)	1636.3 (435.7)	14.8 (−111.1, 140.6)	.818	191.7 (110.7, 272.7)	<.001
tBMD, mg/cm^3^	315.2 (34.0)	276.6 (38.5)	38.6 (27.3, 50.0)	<.001	40.5 (28.8, 52.3)	<.001
Radius pQCT (n = 160)						
Total BA, mm^2^	161.1 (32.5)	161.8 (29.7)	−1.1 (−10.8, 8.57)	.823	7.63 (−1.43, 16.7)	.099
cBMD, mg/cm^3^	1170.0 (38.1)	1151.2 (60.4)	18.8 (3.52, 34.0)	.016	27.1 (10.8, 43.5)	.001
Cortical BA, mm^2^	99.8 (16.7)	96.7 (20.4)	3.10 (−2.68, 8.88)	.293	12.1 (7.32, 16.8)	<.001
SSI, mm^3^	241.1 (63.3)	233.7 (65.9)	7.43 (−12.9, 27.7)	.473	34.7 (17.6, 51.8)	<.001
tBMD, mg/cm^3^	286.9 (34.5)	264.0 (33.9)	22.9 (12.2, 33.7)	<.001	26.7 (14.7, 38.6)	<.001

Abbreviation: cBMD, cortical BMD; sBMD, standardized BMD; tBMD, trabecular BMD; BA, bone area.

a HBM cases are excluding *LRP5* HBM. All pQCT measures were taken from the 66% and 60% slices for tibia and radius, respectively, except for trabecular density measured at the 4% slice.

b Adjusted for age, gender, historical and current PA, height, TB FM, and years since menopause and estrogen replacement therapy in women, with *P* values from regression accounting for within-family clustering.

c Standard range 0–80 pmol/L. Unadjusted median [IQR] for HBM cases and controls: 81.1 [61.6, 103] and 60.4 [43.7, 86] pmol/L, respectively).

d There were145 HBM cases with L1 Z-score ≥+3.2 and mean (SD) sclerostin of 91.1 (40.7) pmol/L; 87 HBM cases with total hip Z-score ≥+3.2 with sclerostin 94.4 (38.2) pmol/L; and 65 HBM cases with both L1 Z-score ≥+3.2 and total hip Z-score ≥+3.2 with sclerostin level of 95.2 (41.6) pmol/L.

e Adjusted for metallic artifact.

**Figure 1.**
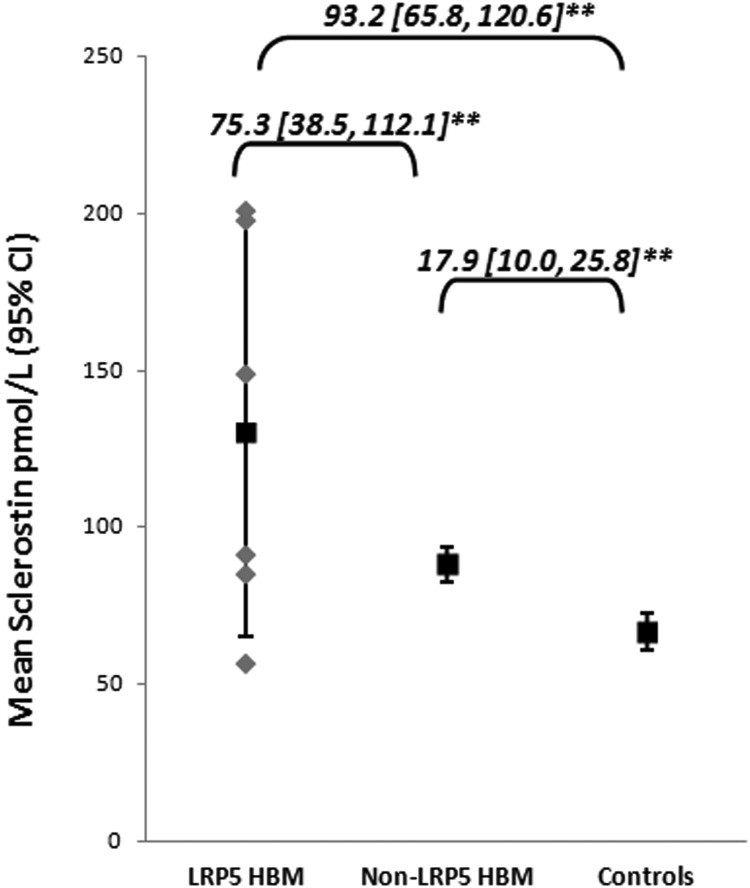
Plasma sclerostin concentrations in HBM cases with and without *LRP5* anabolic mutations and controls. *LRP5* HBM cases n = 6; non-*LRP5* HBM cases n = 196; and controls n = 123. **, *P* < .001. Unadjusted mean difference between *LRP5* HBM cases and controls = 63.4 [35.4, 91.5]**, between non-*LRP5* HBM cases and controls = 22.1 [13.3, 30.9]**, and between *LRP5* HBM cases and non-*LRP5* HBM cases = 42.0 [9.32, 74.8], *P* = .012. Mean differences in sclerostin are shown with 95% CIs after adjustment for a priori confounders (age, gender, historical and current PA, height, TB FM, and years since menopause and estrogen replacement therapy in women). The unadjusted data points for the 6 *LRP5* HBM cases are superimposed in gray rectangles.

To determine the impact of rare cases of HBM caused by anabolic mutations in *LRP5*, identified by previous Sanger sequencing (43), we first assessed sclerostin concentrations in 6 cases of *LRP5* HBM and second in the 196 non-*LRP5* HBM cases. Sclerostin concentrations were highest among the 6 *LRP5* HBM cases (mean [SD], 130.1 [62.7] pmol/L) (Figure 1) but were also elevated in non-*LRP5* HBM cases compared with controls (unadjusted mean difference 22.1 [13.3, 30.9] pmol/L, *P* < .001) (Figure 1). Adjustment for a priori confounders did not diminish the difference in sclerostin concentrations observed between non-*LRP5* HBM cases and controls (Figure 1). The a priori adjusted mean difference in sclerostin levels between *LRP5* and non-*LRP5* HBM cases was halved by further adjustment for TB BMD (mean difference, 35.2 [−0.92, 71.3] pmol/L, *P* = .056).

### Sclerostin and DXA-measured BMD

As previously reported (29), BMD was considerably higher in HBM individuals than controls in unadjusted analyses and persisted after adjustment for a priori and additional confounders (Table 2 and Supplemental Table 1). To establish whether sclerostin differences could be explained by variation in BMD, we first investigated the relationship between DXA BMD and sclerostin in our combined study population. Before adjustment, strong positive relationships were seen between BMD and sclerostin (β represents SD change in sclerostin per SD increase in BMD) measured at L1 (0.32 [0.22, 0.43]), the total hip (0.25 [0.15, 0.35]) and TB (0.26 [0.16, 0.37]) (*P* < .001 for all). Equivalent relationships were observed after adjustment for a priori confounders (Table 3) and additional confounders (Supplemental Table 2). In further analyses, intended to examine whether BMD-sclerostin relationships differed according to HBM case status, associations were generally stronger between DXA BMD parameters and sclerostin in HBM cases, as judged by β-coefficients, especially for L1 BMD; however, despite this, no formal HBM case-control interactions were detected (all *P* > .05) (Table 3).

**Table 3.** Adjusted Regression Coefficients for Associations Between Sclerostin and Standardized Bone Parameters^a^

	β (95% CI)^b^	*P* Value^b^	*P* Value^c^
DXA BMD (n = 320)			
L1 sBMD, g/cm^2^			
All	.290 (.185, .395)	<.001	.167
HBM	.313 (.087, .539)	.007	
Controls	.051 (−.129, .231)	.580	
Total hip sBMD, g/cm^2^			
All	.339 (.203, .448)	<.001	.402
HBM	.341 (.129, .554)	.002	
Controls	.127 (−.104, .358)	.283	
TB BMD,^d^ g/cm^2^			
All	.344 (.223, .465)	<.001	.464
HBM	.310 (.093, .528)	.005	
Controls	.156 (−.044, .356)	.126	
Tibia pQCT (n = 156)			
Total BA, mm^2^			
All	.029 (−.214, .272)	.818	.213
HBM	.042 (−.323, .408)	.821	
Controls	−.180 (−.510, .150)	.286	
Cortical BMD, mg/cm^3^			
All	.182 (.018, .346)	.029	.191
HBM	.061 (−.222, .344)	.673	
Controls	.291 (.083, .499)	.006	
Cortical BA, mm^2^			
All	.451 (.171, .730)	.002	.325
HBM	.268 (−.208, .744)	.270	
Controls	.526 (.107, .944)	.014	
SSI, mm^3^			
All	.323 (.043, .602)	.024	.367
HBM	.172 (−.295, .638)	.471	
Controls	.232 (−.195, .659)	.287	
Trabecular BMD, mg/cm^3^			
All	.307 (.147, .468)	<.001	.301
HBM	.298 (.032, .564)	.028	
Controls	.113 (−.163, .388)	.422	
Radius pQCT (n = 160)			
Total BA, mm^2^			
All	−.060 (−.224, .104)	.476	.140
HBM	−.081 (−.305, .143)	.478	
Controls	−.098 (−.359, .163)	.462	
Cortical BMD, mg/cm^3^			
All	.252 (.101, .402)	.001	.699
HBM	.239 (−.027, .504)	.078	
Controls	.106 (−.098, .310)	.309	
Cortical BA, mm^2^			
All	.254 (.067, .441)	.008	.363
HBM	.147 (−.156, .449)	.342	
Controls	.170 (−.093, .432)	.204	
SSI, mm^3^			
All	.119 (−.067, .305)	.211	.303
HBM	.043 (−.229, .315)	.756	
Controls	.037 (−.245, .318)	.799	
Trabecular BMD, mg/cm^3^			
All	.382 (.228, .537)	<.001	.009
HBM	.499 (.264, .735)	<.001	
Controls	.137 (−.068, .342)	.190	

Abbreviation: sBMD, standardized BMD.

a HBM cases are excluding *LRP5* HBM. All pQCT measures were taken from the 66% and 60% slices for tibia and radius, respectively, except for trabecular density measured at the 4% slice (95 HBM cases and 65 controls). The β-values represent SD change in sclerostin per SD increase in BMD/bone parameter.

b Adjusted for age, gender, historical and current PA, height, TB FM, and years since menopause and estrogen replacement therapy in women.

c Interaction *P* value.

d Adjusted for metal artifact.

### Sclerostin and bone microarchitecture measured by pQCT

The positive relationships observed between DXA-measured BMD and sclerostin were next investigated using lower and upper limb pQCT available in 95 HBM cases and 65 controls (4 tibial pQCT images discarded due to movement artifact). When the clinical characteristics of individuals undergoing pQCT assessment were compared with those who did not, no differences were observed in gender, age, weight, height, physical activity, menopausal status, or estrogen replacement use (data not shown). Before adjustment, trabecular density was markedly greater at both the tibia and radius in HBM cases compared with controls, as were cortical density and thickness, albeit to a lesser extent (Table 2). After adjustment for a priori confounders, trabecular density, cortical density, cortical BA, and SSI, at both the tibia and radius, were all observed to be greater in HBM cases compared with controls; however, bone sizes (total BA) were similar (Table 2). Equivalent results were obtained after adjustment for additional confounders (Supplemental Table 1).

**Figure 2.**
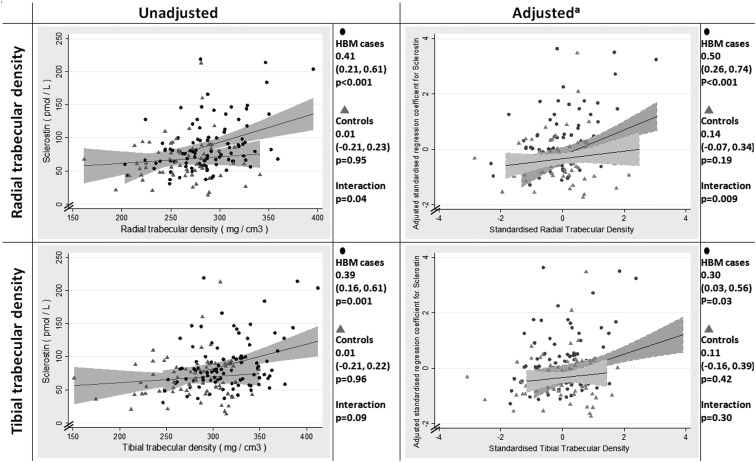
Plasma sclerostin concentrations vs trabecular density measured by pQCT at the distal radius and tibia. HBM represents HBM cases not explained by *LRP5* mutations (●), and FC represents family controls (gray triangles). β represents SD change in sclerostin per SD increase in trabecular density, with 95% CI shown. a, Adjusted for age, gender, historical and current PA, height, TB FM, and years since menopause and estrogen replacement therapy in women.

Using our regression model adjusted for a priori confounders, we assessed the strength of relationships between SD changes in our pQCT measures of bone microarchitecture and sclerostin (standardized). In the study population as a whole, at both the radius and tibia, strong positive relationships were seen between trabecular density, cortical density, cortical BA, and sclerostin; a relationship with SSI was seen only at the radius. Sclerostin was independent of bone size (total BA) in both upper and lower limbs (Table 3). These relationships were unchanged by further adjustment for PINP, plasma CTX, and osteocalcin (data not shown) or by further potential confounders (diabetes mellitus, malignancy, and glucocorticoid and antiresorptive use) (Supplemental Table 2). In stratified analyses, few consistent differences were observed in the relationships between pQCT parameters and sclerostin in HBM cases and controls. The main exception was the association between trabecular density and sclerostin, which was stronger in HBM cases compared with controls at both the radius and tibia, with a formal interaction by case status observed in the upper limb (Table 3 and Figure 2).

### Sensitivity analyses

Relationships between measured (DXA and pQCT) bone parameters and sclerostin were not materially altered after exclusion of *LRP5* HBM cases (Supplemental Tables 3–7).

## Discussion

This study is the first to measure circulating sclerostin concentrations in a large population with HBM. We found HBM cases, identified by screening routine NHS DXA databases across the United Kingdom, to have substantially increased sclerostin concentrations in comparison with their unaffected family members. Sclerostin concentrations in most of our *LRP5* HBM cases, previously identified by capillary sequencing of exons 2, 3, and 4 (43), were higher compared not only with controls but also with the remainder of the HBM population. Our findings are consistent with the one *LRP5* HBM family pedigree in which sclerostin has been measured; although the specific mutation differed from ours, average sclerostin concentrations were almost double that of controls, just as we observed (25). Even after excluding individuals with *LRP5* mutations, sclerostin concentrations were significantly higher in our HBM cases than among controls, a difference unchanged by adjustment for factors we confirmed influence sclerostin concentrations, such as age and gender.

The higher sclerostin concentrations among our HBM cases are likely to be, at least partly, explained by the positive relationship between sclerostin and BMD. This relationship, previously reported in population-based studies of lumbar and total hip BMD (4, 13, 22), was also seen here at L1, total hip, and TB BMD among pooled HBM cases and controls. This may reflect an association between sclerostin and total osteocyte number, given that osteocytes are a major source of sclerostin (44, 45), and BMD reflects the amount of bone tissue and hence osteocyte number. Our microarchitectural analyses support elevated sclerostin concentrations reflecting a greater quantity of bone tissue in HBM cases. As previously reported (26), pQCT analyses revealed HBM cases to have greater cortical and trabecular bone, demonstrated by increased cortical area and trabecular density, respectively, both of which showed positive associations with sclerostin in pooled analyses of HBM cases and controls. These findings concur with recent population-based analyses in which sclerostin has been positively related to cortical bone area and trabecular density in older women (24) and cortical thickness and trabecular density in adult men (23).

We identified a positive relationship between sclerostin and cortical BMD, as observed in population-based studies (23, 24); this may contribute to the increased sclerostin in HBM cases, because cortical BMD is also raised in HBM. Dense cortical bone, with fewer remodeling spaces, may consequently harbor more osteocytes, resulting in greater sclerostin production. However, greater cortical BMD may result in greater measured cortical thickness by reducing the impact of partial volume effects that otherwise limit edge detection accuracy in the presence of low cortical BMD. Alternatively, because cortical BMD is inversely related to bone remodeling and turnover, the positive relationship between sclerostin and cortical BMD, which we and others have observed, may reflect an inverse association between bone turnover and plasma sclerostin. Such a relationship has previously been suggested in postmenopausal women and older men (3, 22), although potentially not for osteocalcin (12), although in the present study, no association was observed between sclerostin concentrations and bone turnover, despite the validity of our sclerostin assay (46). The clinical utility of sclerostin measurement remains to be determined.

Although sclerostin concentrations were elevated in HBM cases both with and without *LRP5* mutations, they were highest in most with *LRP5* mutations compared with other HBM cases. This may reflect a more extreme phenotype in *LRP5* HBM, with greater amounts of bone tissue (reflected by greater trabecular and cortical bone volumes) (26) and hence osteocyte number, than occurs in non-*LRP5* HBM cases. Consistent with this suggestion, *LRP5* HBM cases had greater BMD compared with the remainder of the HBM population (our unpublished observations); *LRP5* HBM mouse models exhibit reduced osteocyte apoptosis (47). Alternatively, individuals with *LRP5* mutations may produce greater amounts of sclerostin for a given quantity of bone tissue compared with non-*LRP5* HBM cases. Although the small numbers of *LRP5* HBM cases limited our ability to examine this question, we found some evidence that HBM cases overall produce relatively large amounts of sclerostin per unit of bone tissue, as reflected by the stronger relationship particularly between radial trabecular density and sclerostin concentrations in HBM cases, than was seen in controls. If HBM cases have predisposing genetic factors toward greater BMD and greater sclerostin concentrations, these effects may be exaggerated in those harboring *LRP5* mutations. For example, rare monogenic *LRP5* HBM cases are likely to have mutations conveying a relatively strong functional effect compared with that of common polymorphisms affecting BMD. Polymorphisms in established BMD genes are known to be overrepresented among individuals with HBM (48–50), suggesting common polymorphisms, each individually exerting relatively weak effects, contribute to the extreme bone phenotype in our non-*LRP5* HBM cases.

Any tendency for sclerostin production to be preferentially increased in *LRP5* HBM may reflect which molecular pathways have been perturbed. LRP5, a cell surface coreceptor regulating canonical Wnt signaling, plays a central role in osteoblast differentiation (51). Anabolic *LRP5* mutations disrupt binding of endogenous Wnt inhibitors such as dickkopf1, prompting activation of downstream signaling and gene transcription via β-catenin. Expression of sclerostin, which also functions as an endogenous inhibitor of Wnt signaling, may conceivably be increased in this context of dysregulated activation of Wnt signaling. Potentially, a subset of non-*LRP5* HBM cases may also arise from genetic perturbations affecting Wnt signaling, which might contribute to the increased sclerostin concentrations observed in our analyses. Interestingly, of the common polymorphisms associated with BMD in large-scale genome-wide association studies, gene ontology links several to roles in osteoblastic Wnt signaling (49, 52); as discussed above, polymorphisms in these BMD-associated loci occur more frequently in our HBM population.

Importantly, sclerostin is not osteocyte-specific; a range of isoforms have been localized in osteoblasts, osteoclasts, and chondrocytes (53). In rodent models, sclerostin is strongly expressed in ossified ligaments and osteophytes emerging by endochondral ossification (37). HBM has been associated with both ligament ossification and increased prevalence of joint replacement (potentially due to osteoarthritis) (29, 54) and, more recently, genetic markers for *MEF2C* and *SOX6,* which both have regulatory roles in endochondral ossification (48, 49).

### Limitations

One potential limitation concerns control individuals comprising relatives/spouses rather than being drawn from the general population. These were considered suitable because 1) they had appropriate BMD (Table 1), 2) they share common environmental factors with cases that would otherwise be difficult to measure and control for as confounding factors, and 3) their inclusion aids future genetic analyses as trait-associated haplotypes can be readily identified. However, family controls are likely to have been more similar to HBM cases than unrelated population controls; hence, clustered analyses were performed to account for the lack of statistical independence due to within-family clustering of environmental factors and shared genotypes. Despite this, our reported differences may still underestimate the true magnitude of the HBM phenotype than had HBM cases been compared with general population controls. We were able to adjust for differences between cases and controls in gender, postmenopausal status, estrogen replacement, glucocorticoid use, and prior history of malignancy, which reflect referral indications for clinical DXA services (29). However, we cannot exclude residual confounding, for example, by PTH or renal function; measurements we lacked. Reduced sample size limited analysis of pQCT measurements that were available in only 50%; however, these individuals were representative of the whole study population.

### Conclusions

Our case-control study found plasma sclerostin concentrations to be increased in HBM cases compared with family controls. These increases were particularly marked in HBM cases with *LRP5* mutations, although cases without *LRP5* mutations also had higher sclerostin concentrations compared with controls. Sclerostin was positively related to BMD, measured by DXA, and to trabecular density and cortical area, measured by pQCT, all of which were measures found also to be increased in HBM. Hence, sclerostin concentrations may be increased in HBM in part due to a greater osteocyte number resulting from greater quantities of trabecular and cortical bone tissue. In addition, greater production of sclerostin per unit of bone tissue may contribute to these differences, as suggested by the stronger relationship between sclerostin concentrations and trabecular density in HBM cases compared with controls. Further analyses of relationships between sclerostin and genetic factors predisposing to HBM is justified to shed new light on the mechanisms regulating sclerostin production.
